# Liver Failure in a Chinese Cystic Fibrosis Child With Homozygous R553X Mutation

**DOI:** 10.3389/fped.2019.00036

**Published:** 2019-02-20

**Authors:** Haiyan Li, Li Lin, Xiaoguang Hu, Changchong Li, Hailin Zhang

**Affiliations:** Department of Pediatric Pulmonology, The Second Affiliated Hospital and Yuying Children's Hospital of Wenzhou Medical University, Wenzhou, China

**Keywords:** cystic fibrosis, CF transmembrane conductance regulator, CF-associated liver disease, liver failure, homozygous CFTR mutation

## Abstract

Cystic fibrosis (CF) is a relatively rare disease in Asians with various clinical characteristics, including CF-associated liver disease (CFLD), which is a common early non-pulmonary complication. This case report describes a Chinese CF patient harboring a homozygous nonsense mutation (c.1657C>T, p.R553X) who was failure to thrive and had intermittently diarrhea during the first year after birth. Liver function test of the patient showed the mildly and intermittently elevated alanine aminotransferase (ALT) levels ranging from 70 to 92 U/L and aspartate aminotransferase (AST) levels ranging from 80 to 90 U/L, which began at 8 months of age and lasted for 4 years without CF diagnosis. In addition, abdominal computed tomography (CT) revealed diffuse fatty infiltration of the liver at 4 years old and gradually developed hepatic cirrhosis. Subsequently, cirrhosis rapidly progressed with obvious splenomegaly and pancreatic insufficiency and the patient died of liver failure with coagulopathy by the age of 6 years old. Pediatricians should remain vigilant to avoid failure to diagnose CF, the occurrence of which may be underestimated, and pay greater attention to the patients with atypical clinical manifestations in Asian countries.

## Introduction

Cystic fibrosis (CF) is an extremely common autosomal recessive genetic disorder with an estimated incidence of 1/25,000 to 1/1,800 among Caucasian populations (1), but relatively rare in the Asian populations (2), especially in China with only a few cases identified in recent years (3). CF often lead to various comorbidities of multiple systems in the body, such as bronchiectasis, small airways obstruction, progressive respiratory impairment, malabsorption, biliary cirrhosis, and infertility (4). Among these comorbidities, CF-associated liver diseases (CFLD) accounts for around 25% of patients with CF (5). Moreover, ~3.3% of mortality makes the CFLD one of the most cause of death in CF (6). However, only 0.9% of all patients experienced liver involvement in the first year of life (7).

Genetic and clinical studies have provided powerful evidence for a definitely causal role for cystic fibrosis transmembrane conductance regulator (CFTR) in the pathogenesis of CF (8, 9). CFTR, as a member of the ATP-binding cassette (ABC) transporter superfamily, functions in controlling ion and water secretion and absorption in epithelial tissues (10). Functional failure of CFTR easily causes multi-system damage in patients with CF (11). Up to now, more than 2,000 different variants have been reported in the CFTR gene available from a database (https://www.cftr2.org/), most of which have been responsible for disease causation (12). The types or regions of CFTR mutation generally vary among patients with CF in different geographical and ethnic origin, may resulting in different effects on the product of protein and affecting processing function or protein stability at the cell membrane (13). Among these different CFTR mutations, although the Phe508del was considered as one of the predominant mutations around the world, it only was a class II traffcking mutation classified by previous studies (4, 14). Other mutation types, such as frameshifts, splicing, and nonsense mutations, may result in no protein production or truncated products, and were categorized as Class I mutations, for example, Trp1282X, Arg553X, and 621+1G>T. These kinds of mutations easily cause more severe phenotype than other types of mutations in patients with CF (14).

Here, we present a case of a Chinese child with CF due to a homozygous nonsense mutation in CFTR, presenting mildly elevated liver enzymes as the initial and main manifestation beginning at the age of 8 months. The patient subsequently developed liver failure induced by cirrhosis and died of coagulopathy at the age of 6 years.

## Case Description

### History

A 4-year-old boy, born in Zhejiang Province of China, was admitted to Yuying Children's Hospital affiliated to Wenzhou Medical University in June 2014 with complaints of productive cough accompanied with high fever for 5 days. He was the first-born child to unrelated healthy parents, born at 38 weeks of gestation after an unremarkable pregnancy. His birth weight was 3.5 kg, and meconium was passed on the first day of life. The patient had no history of meconium ileus or diabetes mellitus and lacked family history of CF. Tracing back his medical history, the patient was formula feeding but failure to thrive with a weight of 6.8 kg at the age of 8 months and had intermittent diarrhea. For further evaluation of the condition of growth and development, the patient was taken to a local hospital at the age of 8 months, and received complete blood count and liver function tests. And the results indicated liver involvement with slightly elevated alanine aminotransferase (ALT) and aspartate aminotransferase (AST) with values of 78 and 82 U/L, respectively. The patient suffered from recurrently and slightly elevated ALT levels ranging from 70 to 92 U/L and AST levels ranging from 80 to 90 U/L. In addition, the common etiologies that easily lead to increased levels of ALT and AST were also excluded, such as cytomegalovirus and hepatitis B virus infection. Initially, these symptoms were not paid enough attention by the physicians or parents because the elevated levels of ALT and AST can recover to normal levels automatically without treatment or through the injection of magnesium isoglycyrrhizinate before four years of age.

### Pathological Findings

**Figure 1:**
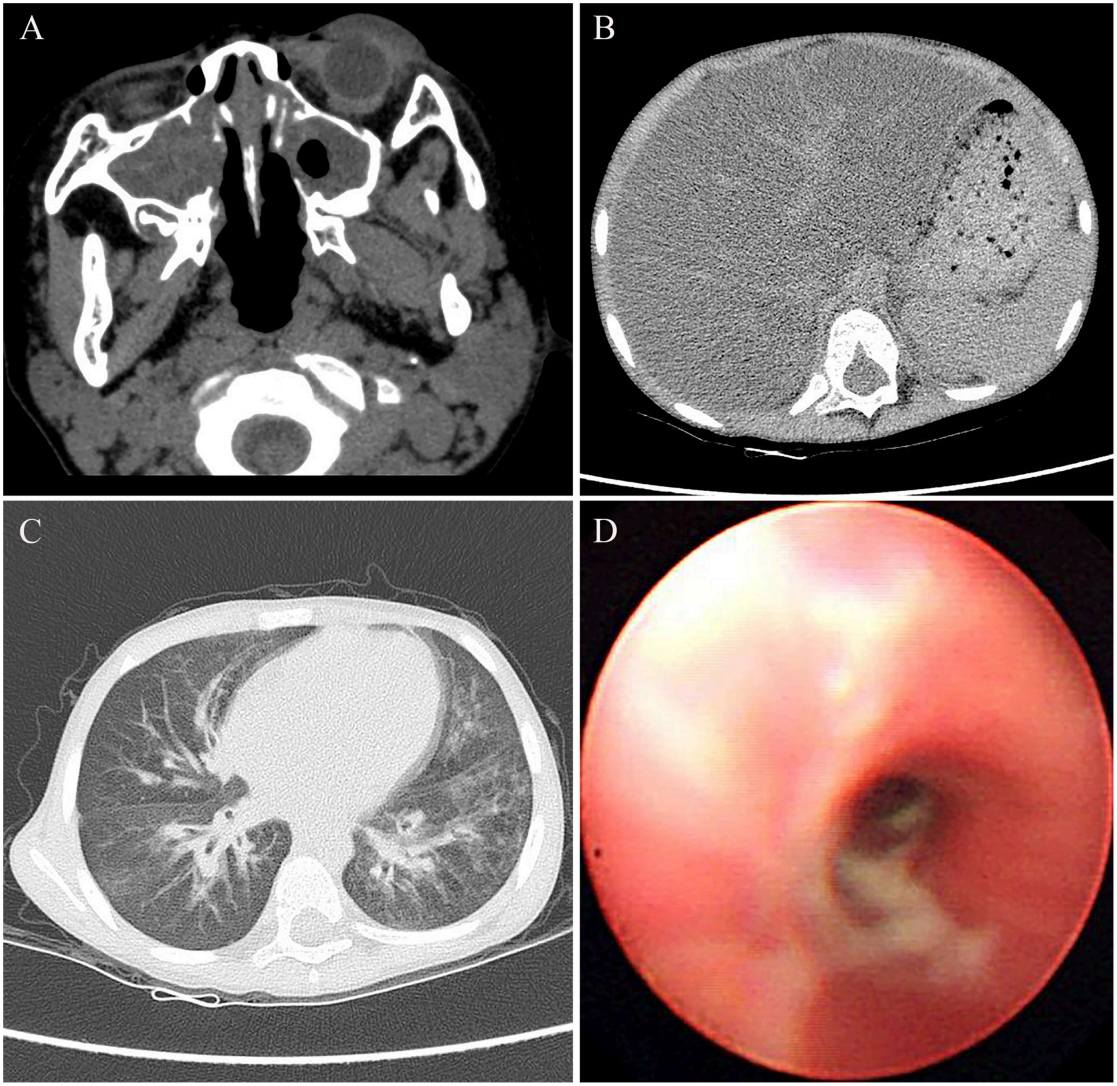
The images of CT and bronchoscopy in 2014, **(A)** maxillary sinuses were almost full of secretions, and **(B)** diffuse liver steatosis was observed. **(C)** Mild bilateral bronchiectasis was observed in CT, and **(D)** purulent secretions were observed in bronchoscopy.

Physical examination for the patient at the age of 4 years showed a weigh of 16.5 kg with a height of 104 cm. The patient had a BMI of 15.3, which was in the 50th percentile for his age. The physical examination also revealed tachypnea and a barrel-shaped chest. The liver was palpable ~2 cm below the right costal margin, and the spleen was palpable about 1 cm below the left costal margin. Clubbed fingers were absent. Laboratory examination indicated increased ALT and AST values of 93 and 92 U/L, respectively, whereas other markers such as γ-glutamyl transferase (GGT), bilirubin, bile acid, fasting blood glucose, albumin, and globulin were within normal limits. Other laboratory investigations including of complete blood count, serum electrolytes, urine, arterial blood gas, amylase, and lipase were normal. The sputum and bronchoalveolar lavage fluid cultures tested positive for *Pseudomonas aeruginosa*. The Sudan III dye test of fecal matter indicated fat droplet positivity. Pulmonary function tests failed to be performed because of the difficulty at this young age for the child. Additionally, the lack of laboratory facilities caused impracticability of the sweat chloride test. Utilizing computed tomography (CT), we identified severe bilateral paranasal sinusitis (Figure 1A) and diffuse fatty infiltration of the liver (Figure 1B) in the patient. In addition, the chest CT scan verified the presence of bilateral bronchiectasis and marked peribronchial thickening, especially in the middle and lower lobes (Figure 1C). Extensive sticky and purulent secretion were observed in the lungs by bronchoscopy (Figure 1D). Based on the aforementioned pathological findings, the patient was primarily diagnosed with CF.

**Figure 2:**
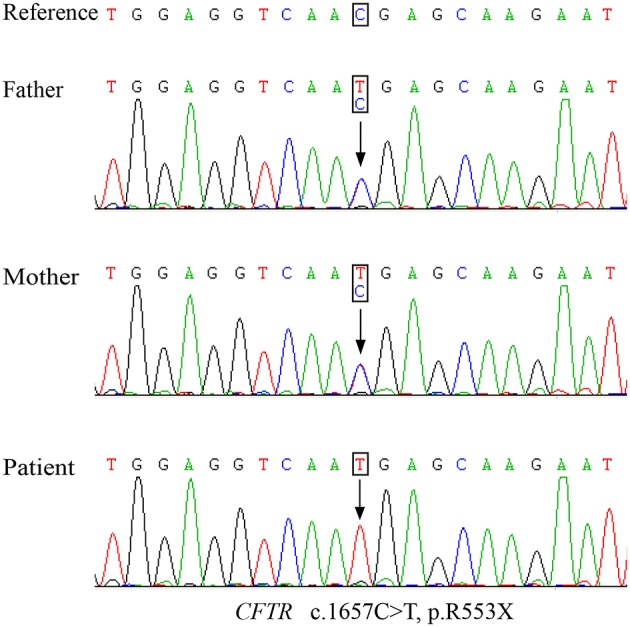
The Sanger sequencing map of the nonsense mutation detected in the parents and patient.

Genetic testing of the patient revealed a homozygous nonsense mutation from a C-to-T substitution (c.1657C > T) in the *CFTR* gene, which was inherited from both his father and mother (Figure 2). This single-nucleotide variant changed an arginine at position 553 into a premature termination codon (p.R553X). Notably, CF screening using amniotic fluid of the mother during her second pregnancy also indicated the fetus (sibling) to be a p.R553X carrier.

### Treatment and Outcome

Other than hypertonic saline nebulization, high-frequency chest wall oscillation, expectorant administration, pancreatic enzyme replacement therapy, and supplementation with vitamins A, D, E, and K, the child was prescribed intravenous ceftriaxone to address the *P. aeruginosa*. Respiratory symptoms gradually improved after 7 days of treatment, and he was discharged on the 15th day after admission. Ursodeoxycholic acid was prescribed after confirmation of genetic diagnosis, but taken irregularly by the patient. Therefore, the medicine failed to bring about the desired effect. Remarkably, the patient was later re-hospitalized two times because of pulmonary infections and liver involvement. Liver function test showed that the levels of both ALT and AST ranged from 90 to 120 U/L. Further examination of abdominal CT and ultrasound have suggested the progression of hepatic cirrhosis. The final hospital admission in August 2016 was due to complaint of a stomachache for 3 days.

**Figure 3:**
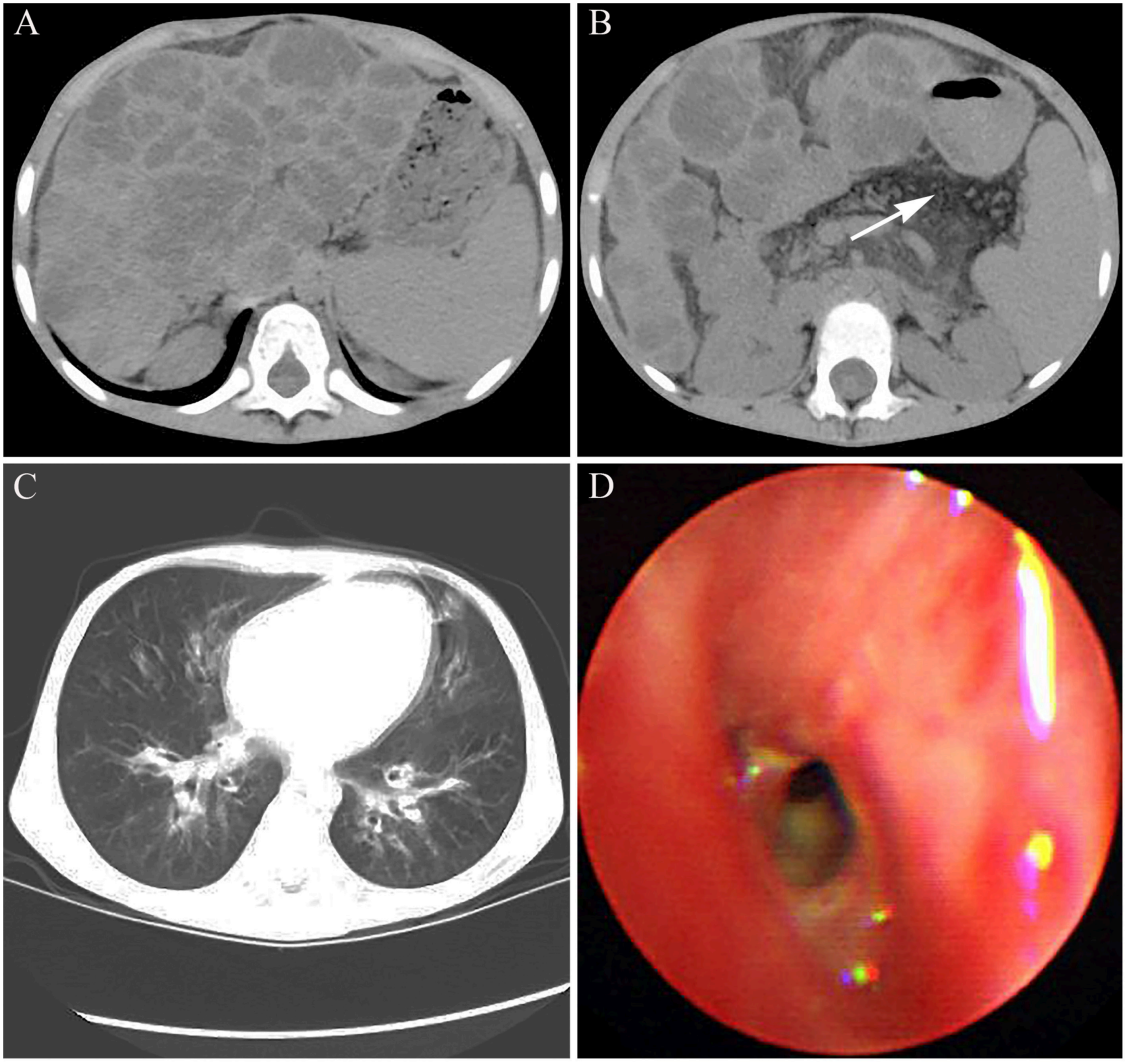
The images of CT and bronchoscopy in 2016. **(A)** enlarged spleen and macronodular cirrhosis on the basis of diffuse hepatic steatosis and **(B)** fatty replacement of the atrophied pancreas indicated by white arrow were observed. **(C)** Mild bilateral bronchiectasis and **(D)** purulent secretions were observed.

Abdominal CT showed a wave-like margin of the liver and many areas of multifocal hypoattenuation in the liver, which indicated the occurrence of hepatic cirrhosis on the basis of diffuse hepatic steatosis (Figure 3A). Simultaneously, the patient presented with pancreatic atrophy and splenomegaly (Figure 3B). In addition, both chest CT scan and bronchoscopy showed the characteristics of bilateral bronchiectasis, marked peribronchial thickening, and extensive sticky and purulent secretion, similar to that observed in 2014 (Figures 3C,D). The abnormal prothrombin time (PT) and activated partial thromboplastin time were 20.2 s (normal control: 13 s) and 52.6 s (normal control: 36 s), respectively. The international normalized ratio (INR) was 1.85, which confirmed the diagnosis of liver failure.

## Discussion

The clinical characteristics of CF in different individuals are various, and liver disease is a relatively common early non-pulmonary complication of CF. It is estimated that 5–10% of CF patients suffer from cirrhosis, often accompanied with portal hypertension (15). In addition, the spectrum of liver involvement, including elevated liver enzymes (ALT, AST, and GGT), hepatic steatosis, focal biliary cirrhosis, multilobular cirrhosis, neonatal cholestasis, and cholangiopathy, has a considerable impact on morbidity and mortality (16). CF is very rarely reported in Chinese people, and thus epidemiological data are lacking. A total of 64 Chinese CF patients with confirmed CFTR mutations have been reported from 1974 up to December 2018, of which only one exhibited the complication of liver cirrhosis (3, 17, 18). These findings were listed in Table 1.

**Table 1:** *CFTR* gene mutations in Chinese CF patients in published literatures.

**Patient**	**cDNA change**	**Amino acid change**	**Gender**	**Family history**	**References**
1	c.1766+5G>T	NA	F	Y	(19)
2	c.1766+1G>T	NA	F	Y	(20)
3	c.1766+5G>T	NA	F	Y	(21)
4	c. 2909G>A, c.319-326delGCTTCCTA	p.G970D, p.A107X	F	N	(22)
5	c.2083dupG, c.2684G>A, c.1766+5G>T	p.E695GfsX35, p.S895N	M	Y	(23)
6	c.2083dupG, c.2684G>A, c.1766+5G>T	p.E695GfsX35, p.S895N	F	Y	(23)
7	c.19G>T, c. 860dupA	p.E7X, p.N287KfsX21	M	Y	(24)
8	c.1766+5G>T, c.2083dupG, c.2684G>A	p.E695GfsX35, p.S895N	F	Y	(24)
9	c.1657C>T	p.R553X	M	N	(25)
10	c.567C>A, c.3691delT	N189K, p.S1231PfsX4	F	N	(26)
11	c.2035-2038? > ?	p.W679X	F	N	(27)
12	c.263T>G, c.2909G>A	p.L88X, p.G970D	F	Y	(28)
13	c.3196C>T	p.R1066C	F	N	(28)
14	c.293A>G	p.Q98R	F	N	(29)
15	c.95T>C, c.1657C>T	p.L32P, p.R553X	M	N	(29)
16	c.293A>G, c.558C>G	p.Q98R, p.N186K	M	N	(29)
17	c.2052 dupA, c.2909-? _3367+? del	p.Q686TfsX3, p.Gly980_ Thr1112delinsGly	M	N	(29)
18	c.2909G>A, c.744-? _1584+? del	p.G970D, p.Arg248_Glu528delinsArgfsX	F	N	(29)
19	c.1666A>G	p.I556V	F	N	(29)
20	c.1679+2T>C, c.2658-1G>C	NA	F	Y^*^	(29)
21	c.214G>A, c.650A>G, c.3406G>A,	p.A72T, p.E217G, p.A1136T,	M	N	(17)
22	c.595C>T	p.H199Y	F	N	(18)
23	c.595C>T, c.2290C>T	p.H199Y, p.R764X	M	N	(18)
24	c.1699G>T, c.3909C>G	p.Asp567Tyr, p.Asn1303Lys	M	NA	(30)
25	c.263T>G, c.1766+5G>T, c.^*^110C>G	p.Leu88X	F	NA	(30)
26	c.3700A>G, c.960_961insA	p.Ile1234Val, p.Ser321IlefsX42	M	NA	(30)
27	c.263T>G, c.2909G>A	p.Leu88X, p.Gly970Asp	F	NA	(30)
28	c.326A>G, c.1000C>T, c.1666A>G	p.Tyr109Cys, p.Arg334Trp, p.Ile556Val	M	NA	(30)
29	c.595C>T	p.His199Tyr	F	NA	(30)
30	c.223C>T, c.326A>G	p.Arg75X, p.Tyr109Cys	F	NA	(30)
31	c.1000C>T	p.Arg334Trp	F	NA	(30)
32	c.263T>G	p.Leu88X	F	NA	(30)
33	c.1666A>G	p.Ile556Val	F	NA	(30)
34	c.293A>G, c.558C>G	p.Gln98Arg, p.Asn186Lys	M	NA	(30)
35	c.326A>G, c.2374C>T	p.Tyr109Cys, p.Arg792X	F	NA	(30)
36	c.1666A>G	p.Ile556Val	M	NA	(30)
37	c.293A>G	p.Gln98Arg	F	NA	(30)
35	c.648G>A, c.2491-126T>C	p.Trp216X	M	NA	(30)
39	c.3196C>T	p.Arg1066Cys	F	NA	(30)
40	c.414_415insCTA	p.Leu138_His139insLeu	M	NA	(30)
41	c.1075C>T, c.3307delA	p.Gln359X, p.Ile1103X	F	NA	(30)
42	c.2909G>A	p.Gly970Asp	F	NA	(30)
43	c.2909G>A, c.1521_1523delCTT	p.G970D, p.F508del	M	N	(31)
44	c.2909G>A, c.2374C>T	p.G970D, p.R792X	F	N	(31)
45	c.2909G>A, c.2125C>T	p.G970D, p.R709X	F	Y	(31)
46	c.3700A>G, c.959–960insA	p.I1234V, p.S321IfsX42	M	N	(31)
47	c.3635delT,	p.V1212AfsX15	M	Y	(31)
48	c.2909G>A, c.1997T>G	p.G970D, p.L666X	F	Y	(31)
49	c.2909G>A, c.263T>G	p.G970D, p.L88X	F	N	(31)
50	c.2909G>A, c.2907A>C	p.G970D, p.A969A	F	N	(31)
51	c.865A>T, c.3651_3652 insAAAT	p.Arg289X, p.Tyr1219X	M	N	(32)
52	c.865A>T, c.3651_3652 insAAAT	p.Arg289X, p.Tyr1219X	M	N	(32)
53	c.3196C>T, c.870-1G>C	p.R1066C	M	NA	(33)
54	c.3G>A, c.1572C>A	p.M1I, p.C524X	F	NA	(33)
55	c.1766+5G>T, c.3068T>G	p.I1023R	M	NA	(34)
56	c.1766+5G>T, c.3140-26A>G	NA	M	NA	(34)
57	c.868C>T, c.3068T>G	p.Q290X, p.I1023R	M	NA	(34)
58	c.1657C>T, c.3068T>G	p.R553X, p.I1023R	F	NA	(34)
59	c.3068T>G, c.3068T>G	p.I1023R	F	NA	(34)
60	c.579+1_579+2insACAT, c.1766+5G>T	NA	M	N	(3)
61	c.595C>T	p.H199Y	M	N	(3)
62	c.1117-1G>C, c.2909G>A	p.G970D	F	N	(3)
63	c.4056G>C	p.Q1352H	M	N	(3)
64	c.263T>G, c.2335C>T	p.L88X, p.Q779X	F	N	(35)
65	c.1657C>T	p.R553X	M	N	This study

* *means the patient's older sister died at the age of 11 as a result of pneumonia. F, female; M, male; NA, not available*.

During the first year after birth, our patient underwent poor growth, malnutrition with a weight of 6.8 kg at 8 months old and intermittent diarrhea without cholestasis and history of meconium ileus. In order to evaluate the condition of growth and development, liver function test was performed and presented mildly and intermittently elevated levels of both AST and ALT from 8 months old to 4 years old. Subsequently, the abdominal CT showed the occurrence of hepatic cirrhosis which rapidly developed with severe portal hypertension and decompensated liver function. The patient died of liver failure with coagulopathy at the age of 6 years. The CT studies of the upper abdomen revealed steatohepatitis in 2014, which developed to cirrhosis based on steatosis with obvious pancreatic atrophy in 2016. The patient suffered from bronchiectasis, but no significant change was found in the chest CT between 2014 and 2016. Although we did not obtain results of the sweat test, a previous study identified that several damaging *CFTR* mutations, including homozygous F508del/F508del mutations, were associated with high sweat chloride levels exceeding 60 mEq/L (36). Therefore, the sweat test is an important tool for CF diagnosis, particularly in the absence of the identification of mutations in the *CFTR* gene.

The *CFTR* gene mutation spectrum of CF has been well established among Caucasian populations. Among over 2,000 mutations within CF, the p.F508del mutation is the most frequent in many western countries including France and Germany (37, 38). A follow-up study reported that male sex, a history of meconium ileus, and severe mutations including p.F508del, c.1717-1G > A, p.G542X, and p.N1303K were considered independent risk factors for CFLD (39). However, a previous study suggested that the development of liver disease in CF was unlikely to be associated with a specific mutation such as p.F508del, p.G551D, or p.R553X (40). In this study, we identified a homozygous p.R553X mutation in a patient who suffered from severe CFLD and died of liver failure. In addition, previous case reports found two CF cases in Chinese harboring compound heterozygous CFTR mutations containing p.R553X and these two cases showed pancreatic exocrine insufficiency and recurrent pneumonia combining with significant bronchiectasis, respectively (29, 33–35). Interestingly, one CF case with homozygous R553X mutation was also reported in Taiwan and developed bronchiectasis with chronic hypoxemia and pancreatic insuffciency, but no liver failure (25, 33, 35). These case reports also indicated the presence of noticeable differences in the *CFTR* gene mutation spectrum between Chinese and Caucasian CF cases (3). Therefore, it is critical to investigate the gene mutation spectrum and relationship between the Chinese genotypes and phenotypes such as CFLD through future investigations.

## Concluding Remarks

In China, patients with CF previously were often misdiagnosed or received a delayed diagnosis. With the development of genetic diagnostic technology, increased cases of CF have been confirmed and reported in China. Pediatricians should remain alert to undiagnosed CF in patients with atypical and nonspecific features such as recurrent respiratory infections, steatorrhea, and mildly elevated AST and ALT without definite causes, which tend to be neglected and lead to diagnostic dilemmas in Asian countries lacking neonatal screening. In this report, the sibling of the patient was a carrier of the heterozygous mutation of p.R553X based on prenatal screening. Therefore, early diagnosis, specialized CF center care, and genetic counseling are critical to improve the prognosis of the disease.

## Ethics Statement

Since this is a case report, no protocol or ethics committee was utilized for this report. Written informed consent for the publication of this case report and figures were obtained from the parents.

## Author Contributions

HL and LL collected data and wrote the manuscript. XH, CL, and HZ reviewed the article.

### Conflict of Interest Statement

The authors declare that the research was conducted in the absence of any commercial or financial relationships that could be construed as a potential conflict of interest.

## References

[1] Colombo C Littlewood J. The implementation of standards of care in Europe: state of the art. J Cyst Fibros. (2011) 10 (Suppl. 2):S7–15. 10.1016/S1569-1993(11)60003-9 21658645

[2] Singh M Rebordosa C Bernholz J Sharma N. Epidemiology and genetics of cystic fibrosis in Asia: in preparation for the next-generation treatments. Respirology (2015) 20:1172–81. 10.1111/resp.12656 26437683

[3] Xu J Yin Y Zhang L Zhang J Yuan S Zhang H. Four case reports of Chinese cystic fibrosis patients and literature review. Pediatr Pulmonol. (2017) 52:1020–8. 10.1002/ppul.23744 28608624

[4] Elborn JS. Cystic fibrosis. Lancet (2016) 388:2519–31. 10.1016/S0140-6736(16)00576-6 27140670

[5] Palaniappan SK Than NN Thein AW Moe S van Mourik I. Interventions for preventing and managing advanced liver disease in cystic fibrosis. Cochrane Database Syst Rev. (2017) 8:CD012056. 10.1002/14651858.CD012056.pub2 28850173 PMC6483789

[6] van de Peppel IP Bertolini A Jonker JW Bodewes F Verkade HJ. Diagnosis, follow-up and treatment of cystic fibrosis-related liver disease. Curr Opin Pulm Med. (2017) 23:562–9. 10.1097/MCP.0000000000000428 28837442

[7] Wagener JS Woo MS Pasta DJ Konstan MW Morgan WJ Investigators and Coordinators of Epidemiologic Study of Cystic Fibrosis Liver involvement in the Hispanic population of North America with cystic fibrosis. J Pediatr Gastroenterol Nutr. (2014) 59:476–9. 10.1097/MPG.0000000000000448 24897167

[8] Riordan JR Rommens JM Kerem B Alon N Rozmahel R Grzelczak Z. Identification of the cystic fibrosis gene: cloning and characterization of complementary DNA. Science (1989) 245:1066–73. 2475911 10.1126/science.2475911

[9] Kerem B Rommens JM Buchanan JA Markiewicz D Cox TK Chakravarti A. Identification of the cystic fibrosis gene: genetic analysis. Science (1989) 245:1073–80. 10.1126/science.2570460 2570460

[10] Quinton PM. Physiological basis of cystic fibrosis: a historical perspective. Physiol Rev. (1999) 79:S3–22. 10.1152/physrev.1999.79.1.S3 9922374

[11] Farrell PM Rosenstein BJ White TB Accurso FJ Castellani C Cutting GR. Guidelines for diagnosis of cystic fibrosis in newborns through older adults: cystic fibrosis foundation consensus report. J Pediatr. (2008) 153:S4–14. 10.1016/j.jpeds.2008.05.005 18639722 PMC2810958

[12] Castellani C Cuppens H Macek M Jr Cassiman JJ Kerem E Durie P. Consensus on the use and interpretation of cystic fibrosis mutation analysis in clinical practice. J Cyst Fibros. (2008) 7:179–96. 10.1016/j.jcf.2008.03.009 18456578 PMC2810954

[13] Dawson KP Frossard PM. The geographic distribution of cystic fibrosis mutations gives clues about population origins. Eur J Pediatr. (2000) 159:496–9. 10.1007/s004310051317 10923221

[14] Wilschanski M Zielenski J Markiewicz D Tsui LC Corey M Levison H. Correlation of sweat chloride concentration with classes of the cystic fibrosis transmembrane conductance regulator gene mutations. J Pediatr. (1995) 127:705–10. 10.1016/S0022-3476(95)70157-5 7472820

[15] Ye W Narkewicz MR Leung DH Karnsakul W Murray KF Alonso EM. Variceal hemorrhage and adverse liver outcomes in patients with cystic fibrosis cirrhosis. J Pediatr Gastroenterol Nutr. (2018) 66:122–7. 10.1097/MPG.0000000000001728 28906321 PMC5745284

[16] Leung DH Narkewicz MR. Cystic fibrosis-related cirrhosis. J Cyst Fibros. (2017) 16 (Suppl. 2):S50–61. 10.1016/j.jcf.2017.07.002 28986027

[17] Li L Wang NL Gong JY Wang JS. [Infantile cholestasis caused by CFTR mutation: case report and literature review]. Zhonghua Er Ke Za Zhi. (2016) 54:851–5. 10.3760/cma.j.issn.0578-1310.2016.11.013. 27806795

[18] Xu BP Wang H Zhao YH Liu J Yao Y Feng XL. [Molecular diagnosis of two Chinese cystic fibrosis children and literature review]. Zhonghua Er Ke Za Zhi. (2016) 54:344–8. 10.3760/cma.j.issn.0578-1310.2016.05.007 27143075

[19] Wang MC Shu SG Chang SM Ho WL Chi CS. Cystic fibrosis in two Chinese infants in Taiwan. Zhonghua Min Guo Xiao Er Ke Yi Xue Hui Za Zhi. (1993) 34:314–21. 8213163

[20] Crawford J Labrinidis A Carey WF Nelson PV Harvey JS Morris CP. A splicing mutation (1898 + 1G—->T) in the CFTR gene causing cystic fibrosis. Hum Mutat. (1995) 5:101–2. 10.1002/humu.1380050115 7537147

[21] Zielenski J Markiewicz D Lin SP Huang FY Yang-Feng TL Tsui LC. Skipping of exon 12 as a consequence of a point mutation (1898 + 5G—->T) in the cystic fibrosis transmembrane conductance regulator gene found in a consanguineous Chinese family. Clin Genet. (1995) 47:125–32. 10.1111/j.1399-0004.1995.tb03944.x 7543385

[22] Wagner JA Vassilakis A Yee K Li M Hurlock G Krouse ME. Two novel mutations in a cystic fibrosis patient of Chinese origin. Hum Genet. (1999) 104:511–5. 10.1007/s004390050996 10453741

[23] Wu CL Shu SG Zielenski J Chiang CD Tsui LC. Novel cystic fibrosis mutation (2215insG) in two adolescent Taiwanese siblings. J Formos Med Assoc. (2000) 99:564–7. 10925568

[24] Alper OM Shu SG Lee MH Wang BT Lo SY Lin KL. Detection of novel CFTR mutations in Taiwanese cystic fibrosis patients. J Formos Med Assoc. (2003) 102:287–91. 12874665

[25] Chen HJ Lin SP Lee HC Chen CP Chiu NC Hung HY. Cystic fibrosis with homozygous R553X mutation in a Taiwanese child. J Hum Genet. (2005) 50:674–8. 10.1007/s10038-005-0309-x 16283068

[26] Li N Pei P Bu DF He B Wang GF. A novel CFTR mutation found in a Chinese patient with cystic fibrosis. Chin Med J. (2006) 119:103–9. 10.1097/00029330-200601020-00003 16454991

[27] Cheng Y Ning G Song B Guo YK Li XS. A Chinese girl with cystic fibrosis: a case report identified by sweat and genetic tests. Chin Med J. (2012) 125:719. 22490504

[28] Liu JR Peng Y Zhao YH Wang W Guo Y He JX. [Clinical manifestations and gene analysis of 2 Chinese children with cystic fibrosis]. Zhonghua Er Ke Za Zhi. (2012) 50:829–33. 23302613

[29] Liu Y Wang L Tian X Xu KF Xu W Li X. Characterization of gene mutations and phenotypes of cystic fibrosis in Chinese patients. Respirology (2015) 20:312–8. 10.1111/resp.12452 25580864

[30] Shen Y Liu J Zhong L Mogayzel PJ Jr Zeitlin PL Sosnay PR. Clinical phenotypes and genotypic spectrum of cystic fibrosis in chinese children. J Pediatr. (2016) 171:269–76 e1. 10.1016/j.jpeds.2015.12.025 26826884

[31] Tian X Liu Y Yang J Wang H Liu T Xu W p.G970D is the most frequent CFTR mutation in Chinese patients with cystic fibrosis. Hum Genome Var. (2016) 3:15063 10.1038/hgv.2015.63 27081564 PMC4785583

[32] Xie Y Huang X Liang Y Xu L Pei Y Cheng Y. A new compound heterozygous CFTR mutation in a Chinese family with cystic fibrosis. Clin Respir J. (2017) 11:696–702. 10.1111/crj.12401 26471113

[33] Zheng B Cao L. Differences in gene mutations between Chinese and Caucasian cystic fibrosis patients. Pediatr Pulmonol. (2017) 52:E11–4. 10.1002/ppul.23539 27717243 PMC5324682

[34] Leung GK Ying D Mak CC Chen XY Xu W Yeung KS. CFTR founder mutation causes protein trafficking defects in Chinese patients with cystic fibrosis. Mol Genet Genomic Med. (2017) 5:40–9. 10.1002/mgg3.258 28116329 PMC5241212

[35] Wang YT Liu JH Yang XD Yan H Zhang YG Chinese data of the CFTR mutation: a report from West China Hospital and literature review. Int J Clini Exp Med. (2018) 11:6293–301.

[36] Faria AG Marson FAL Gomez CCS Servidoni MF Ribeiro AF Ribeiro JD. Thirty years of sweat chloride testing at one referral center. Front Pediatr. (2017) 5:222. 10.3389/fped.2017.00222 29124052 PMC5662556

[37] Guilloud-Bataille M De Crozes D Rault G Degioanni A Feingold J. Cystic fibrosis mutations: report from the French registry. The clinical centers of the CF. Hum Hered. (2000) 50:142–5. 10.1159/000022903 10799974

[38] Tummler B Storrs T Dziadek V Dork T Meitinger T Golla A. Geographic distribution and origin of CFTR mutations in Germany. Hum Genet. (1996) 97:727–31. 10.1007/BF02346181 8641688

[39] Colombo C Battezzati PM Crosignani A Morabito A Costantini D Padoan R. Liver disease in cystic fibrosis: a prospective study on incidence, risk factors, and outcome. Hepatology (2002) 36:1374–82. 10.1002/hep.1840360613 12447862

[40] Duthie A Doherty DG Williams C Scott-Jupp R Warner JO Tanner MS. Genotype analysis for delta F508, G551D and R553X mutations in children and young adults with cystic fibrosis with and without chronic liver disease. Hepatology (1992) 15:660–4. 10.1002/hep.1840150418 1551644

